# Various neuromodulation methods including Deep Brain Stimulation of the medial forebrain bundle combined with psychopharmacotherapy of treatment-resistant depression—Case report

**DOI:** 10.3389/fpsyt.2022.1068054

**Published:** 2023-01-16

**Authors:** Joanna Rymaszewska, Tomasz Wieczorek, Karolina Fila-Witecka, Katarzyna Smarzewska, Artur Weiser, Patryk Piotrowski, Paweł Tabakow

**Affiliations:** ^1^Department of Psychiatry, Wroclaw Medical University, Wrocław, Poland; ^2^Department of Neurosurgery, Wroclaw Medical University, Wrocław, Poland

**Keywords:** treatment-resistant depression, neurostimulation, Deep Brain Stimulation, Transcranial Magnetic Stimulation, electroconvulsive therapy

## Abstract

**Background:**

Treatment-resistant depression remains one of the main concerns of modern psychiatry. Novel methods such as Transcranial Magnetic Stimulation (including deep and theta burst protocols, iTBS) and Deep Brain Stimulation (DBS) can be considered as alternative treatment options.

**Case presentation:**

Twenty-nine-year-old Caucasian female, single, higher-educated was treated with major depressive disorder initially with standard pharmaco- and psychotherapy. Due to diagnosed treatment resistance additional therapeutic approaches were introduced sequentially: Electroconvulsive therapy (efficient only 4 months) and Transcranial Magnetic Stimulation (intermittent Theta Burst Stimulation, iTBS improved just insomnia). Finally the patient was enrolled to the Deep Brain Stimulation (DBS) study with the medial forebrain bundle target. After 20 months of active DBS a reduction of over 80% of depressive symptom severity was observed (Montgomery-Asberg and Hamilton Depression Rating Scales), together with an 87% reduction of anxiety symptoms intensity (Hamilton Anxiety Rating Scale) and a 90% increase in social and occupational functioning. Subjective assessment of the patient performed with questionnaires and visual analog scales showed less pronounced improvement in terms of depressive and anxiety symptoms, and high reduction of anhedonia. Some mild, transient side effects of neurostimulation were eliminated with an adjustment in stimulation parameters.

**Conclusions:**

The presented clinical case confirms the possibility of achieving remission after the use of MFB DBS in treatment-resistant depression, but postponed for many months. Nevertheless, personalization of every combined therapy with DBS is necessary with exploration of individual factors as past traumas and personality traits. More reports on long-term observations in DBS treatment in TRD trials (especially focused on MFB target) are needed.

## 1. Introduction

**Figure 1:**
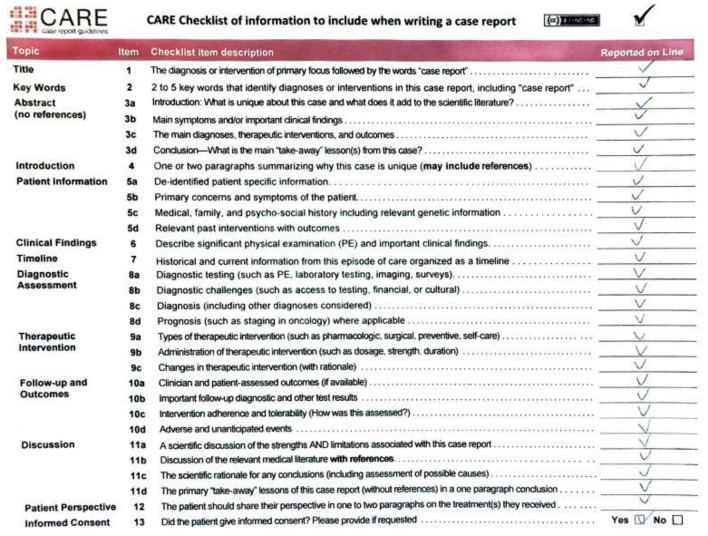
CARE checklist.

Treatment-resistant depression (TRD) has become one of the major concerns of psychiatry nowadays. Pharmacotherapy, psychotherapy and electroconvulsive therapy are usually used as standard treatment options for, however not all of the patients respond to these methods, even when combined (1, 2). Novel methods such as Transcranial Magnetic Stimulation (including deep and theta burst protocols, iTBS) and Deep Brain Stimulation (DBS) can be considered as alternative treatment options for TRD patients (2–4). Moreover, TRD comorbid with combined personality traits and trauma history might be still considered as a demanding clinical challenge. A case of a complex clinical problem with all mentioned therapeutic options is presented. The study protocol was approved by a Bioethical Committee at the Wroclaw Medical University (No. KB-363/2017). The patient signed a permission for publication. All performed clinical evaluations have been video-recorded after a written consent. CARE checklist was filled after the manuscript has been written (see Figure 1).

## 2. Case description

We report a case of a 29-year-old Caucasian female, single, higher-educated, without any significant somatic comorbidities, and employed as an accountant.

### 2.1. Medical, family, and psychosocial history

Her grandfather committed suicide at the age of 34, the father is alcohol-dependent. During her childhood the patient describes her parents as largely neglectful toward her with a lot of domestic quarreling, which took place in her presence. The relationship with her father was weak and distant and her mother was controlling and emotionally labile with occasional angry outbursts toward the patient. Her parents divorced when she was 22 years old. The patient decided not to pursue the relationships with them as an adult. As a teenager she experienced an incident of sexual abuse at the hands of a peer, who also stalked her for some time after the assault. There is a negative history for psychoactive substance use, somatic illnesses, and positive for self-harm in high school (cutting her skin).

### 2.2. Past interventions and outcomes

The patient first sought psychological help at the age of 17 the onset of major depressive disorder (MDD), after a self-harm episode, when the school intervened and informed the parents. She remained in psychotherapy for around 4 years, assessing it as effective. In that time, she was able to finish college, start working in her profession as well as keep up with several hobbies and stay very active most of the time.

Since 2018 the patient experienced a slow decline in her mental wellbeing, with no visible cause or significant life events that may have contributed. Since May 2018 the patient continued psychological treatment in the form of counseling and individual psychotherapy, neither yield any significant improvements in the patient's mood and were overall considered ineffective by her. Once the psychotherapy was terminated by the therapist due to worsening of mental health status (suicidal ideation) and inpatient treatment was recommended instead. Before enrolment in the study the patient had been hospitalized two times in stationary wards (one stay after a suicidal attempt in March 2019) and three times in daily wards, diagnosed with MDD with avoidant personality disorder. In the course of her treatment, dominant MDD symptoms were: depressed mood, lack of motivation, decreased complex activity level, anhedonia, and insomnia.

#### 2.2.1. Pharmacotherapy

Pre-DBS pharmacotherapy consisted of sertraline, mirtazapine (up to 45 mg), venlafaxine (up to 225 mg), bupropion (up to 300 mg), quetiapine (up to 150 mg), escitalopram (up to 20 mg), and vortioxetine (up to 20 mg) administered for pharmacokinetically adequate periods. In longer observations the best self-reported functioning was during bupropion intake, however none of pharmacological agents provided stable remission or significant improvement in terms of MDD symptoms and functioning.

#### 2.2.2. Electroconvulsive therapy (ECT)

During the hospitalization in November 2019–January 2020 (admitted from the toxicology department after her second suicidal attempt) one course of ECT was performed in the Psychiatry Clinic of the Wroclaw University Hospital. Titration method was used with a total number of eight successful treatment sessions. Final parameters were as follows: frequency = 35 Hz, bandwidth = 1 ms, current = 800 mA, and stimulation duration = 4 s. During the treatment the patient reported teeth aches with no other side effects. Bilateral temporal areas were stimulated with manual electrodes. During each stimulation provoked seizure attack lasted at least 25 s assessed both by EEG and muscle contraction sensor placed on pointing finger not blocked by suxamethonium chloride. Since ECT therapy was implemented before enrolment to the DBS study none of precise assessment tools were used apart from a brief clinical global impression scale (CGI) assessment.

After the ECT treatment in parallel with the psychopharmacotherapy (bupropion 150 mg, quetiapine 150 mg) and psychological support a remission of MDD symptoms was observed. Pharmacotherapy during the discharge consisted of quetiapine 150 mg, bupropion 300 mg, and propranolol 30 mg. During subsequent months she returned to full-time job and took part in a TV-video documentary about ECT treatment that was recorded in the Clinic.

Four months after ECT a gradual relapse of MDD symptoms was observed. In July 2020 escitalopram (20 mg) was added to bupropion and quetiapine with no significant improvement. After being presented with various treatment options, including maintenance ECT, the patient did not want to take electroconvulsive therapy again. The patients' concern was memory impairment after repeated ECT courses. Even if transient, this would strongly interfere with her work skill and responsibility as an accountant.

#### 2.2.3. Transcranial Magnetic Stimulation (TMS)

In September 2020 the patient was recruited to the clinical program of TMS treatment in MDD. It was a randomized sham-controlled study comparing a “classical” FDA-approved rTMS protocol for MDD with an authored intermittent theta-burst stimulation (iTBS) protocol. The patient was randomized into the active iTBS protocol, consisting of a total number of 40 sessions (4 daily), 189 s each, 80% of RMT, 50 Hz. Self-reported improvement of sleep duration and its quality was reported, but only a relatively small reduction of MDD symptoms and no change in functioning were observed after the TMS treatment. Due to the sudden exacerbation of depressive symptoms and appearance of intense suicidal ideations at 9th day of the stimulation, it was ceased. Thus, only 32 out of total 40 sessions protocol-wise were performed. The sudden exacerbation od MDD symptoms in the authors' opinion was not due to TMS stimulation.

### 2.3. Therapeutic intervention: Deep Brain Stimulation

At the end of October 2020 the patient was recruited to the study of DBS treatment in TRD. In this time the patient met all the inclusion criteria and there were no exclusion criteria present (the suicidal ideations subsided), presented in Table 1. Meanwhile pharmacotherapy was modified—bupropion, quetiapine and escitalopram were withdrawn, for a short period of time amitriptyline (150 mg) was introduced and fast withdrawn due to severe side effects (urinary retention). Vortioxetine (10 mg) and mirtazapine (30 mg) were introduced with partial positive response in terms of sleep quality.

**Table 1:** Inclusion and exclusion criteria of the DBS in TRD study.

**Inclusion criteria**	**Exclusion criteria**
Resistance to previous treatment (lack or insufficiency of effect): — three courses of treatment with anti-depressive drugs in adequate doses for at least 6 weeks—including at least one treatment with serotonin and norepinephrine reuptake inhibitor (SNRI) or tricyclic anti-depressant; — one adjuvant therapy with lithium or second generation anti-psychotic drug for at least 6 weeks; — at least one course of CBT or interpersonal therapy (at least 16 therapeutic sessions); — one electroconvulsive therapy course or contraindications for this treatment.	Comorbidities: — mental disorders (psychotic disorders, bipolar disorder, autism, severe personality disorders, psychoactive substance addiction, and dementia), — unstable somatic state, — disorders of CNS (including epilepsy, PD, and multiple sclerosis)
Diagnosis of MDD: — confirmed according to DSM-V criteria; — score of at least 20 points in Hamilton Depression Rating Scale-17 (HDRS-17), — GAF score <50 points, — at least 5 years of MDD duration	Presence of some MDD-related symptoms: — auto aggressive behaviors, — self-injuries, — present risk of suicidal act, — unstable and severely impaired functioning
Age in range from 18 to 65 years	•Pregnancy
IQ more than 80	

In December 2020 a DBS system was implanted bilaterally into the medial forebrain bundle (MFB) area, according to the technique described by Coenen et al. (5). Two four-contact directional leads (1.5 mm length of each contact and 0.5 mm interspacing), connected to a rechargeable stimulator (Gevia, Boston, USA), were implanted. Stimulation parameters were: 130 Hz, 60 μs and a maximal amplitude of 3 mA. Both bipolar and directional monopolar (cathodal and anodal) were used throughout the study. In January 2021 the stimulation was initiated and a sudden exacerbation of MDD symptoms within 2 weeks was observed. After the adjustment of stimulation parameters and increase of vortioxetine dose to 20 mg an almost total cessation of depressive and “internal tension” symptoms was observed.

**Figure 2:**
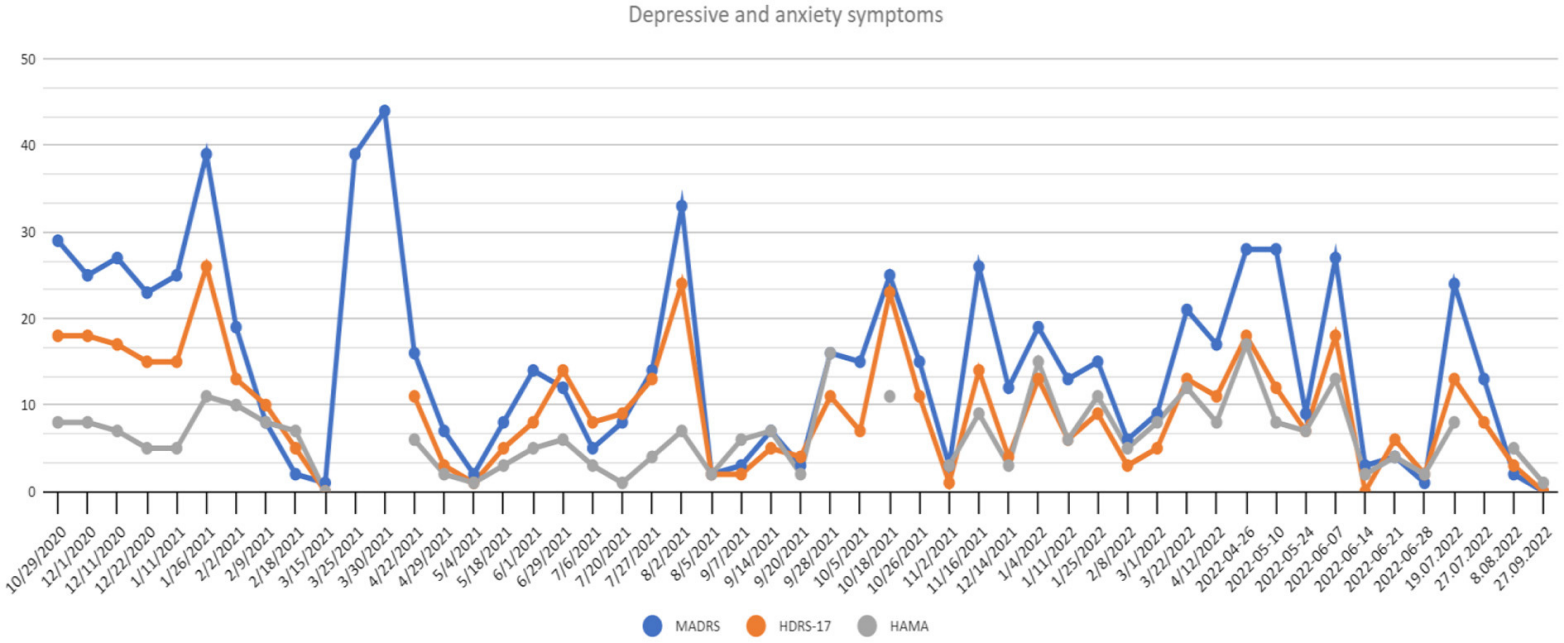
Depressive and anxiety symptoms scores at recruitment point and during 20 months of active DBS stimulation period. HAMA, Hamilton Anxiety Rating Scale; HDRS-17, Hamilton Depression Rating Scale 17-item version; MADRS, Montgomery-Asberg Depressive Rating Scale.

In the middle of March 2021 the patient reached full remission of symptoms (defined as HDRS ≤ 7/MADRS ≤ 10 scores). The patient also reported improvement in self-assessment VAS scales (see Figure 2).

Unfortunately, the patient got infected with SARS-CoV-2 (mild severity) and was isolated alone at home. Immediately after coming back to work her MDD symptoms intensified rapidly. At the end of March the patient was admitted to toxicology and then psychiatric ward after a suicidal medication overdose with mirtazapine (1,200 mg) and chlorprothixene (300 mg).

During her stay in the Psychiatry Clinic vortioxetine 20 mg and active DBS were continued. Significant improvement was observed after lithium carbonate introduction (3 × 250 per day, orally). However, due to past suicidal attempts and possible risk of such acts in the future finally the stabilizer was changed into lamotrigine (150 mg) and the patient was discharged. Depressive symptoms were subsiding further, a relatively stable 4 months remission was observed until the end of July.

Following the study protocol an attempt of parameter modification was performed in order to verify if the improvement could be attributed to DBS. The patient and the rating psychiatrists remained blinded, while the neurosurgeons were unblinded to introduced modifications. A sudden relapse of symptoms with subjective worsening of mental state (see Figure 2) and stimulation side effects were reported (hand tremors, tachycardia, vision blurring and diplopia). After rehospitalization and the adjustment of parameters again the fast MDD remission was achieved with good treatment tolerance.

In mid-October 2021 an exacerbation of MDD symptoms was observed, which could be explained by the increased impedance of C6 electrode revealed during check-up. Cessation of stimulation in this area was performed with following fast remission over the next 2 weeks. In mid-November a relapse appeared with no determined cause and the patient asked for the change of pharmacotherapy. It was decided that lithium carbonate (500 mg, plasma level 0.59 mmol/l) and bupropion (300 mg) will be reintroduced additionally to vortioxetine (10 mg) whereas lamotrigine will be withdrawn. A significant response was observed.

In January 2022 the patient finished 1st year of active DBS stimulation with improvement in the severity of MDD symptoms and functioning. But starting from March 2022, sleep disturbances and insomnia symptoms appeared, with only partial response to pharmacotherapy changes (firstly doxepine 10 mg instead of chlorprothixene, but with no effect; then promazine 25 mg was introduced with quetiapine increase to 100 mg) and starting from April 2022 an exacerbation of MDD symptoms appeared with no significant stressors appearing in the patient's life. Due to the high level of anxiety, temporary reintroduction of clonazepam 1 mg daily was necessary. An improvement was observed, but together with clonazepam dose reduction, another decline was observed.

At the beginning of June 2022 DBS parameters were changed for those used during the period from February to July 2021 with one modification—the cathode was set on the internal pulse generator, and anodic stimulation at the level of the electrodes was introduced. Simultaneously, vortioxetine was increased to 20 mg, lithium carbonate decreased to 250 mg and lamotrigine 50 mg was reintroduced. A fast improvement was observed, but lasting only for 1 month, and in the middle of July DBS was changed from anodic current to bipolar, with parameters similar to those used in February–July 2021, with an exception of one contact remaining turned off due to a high impedance level. No abnormalities were observed in physical examinations and laboratory tests performed each time during hospitalization.

**Figure 3:**
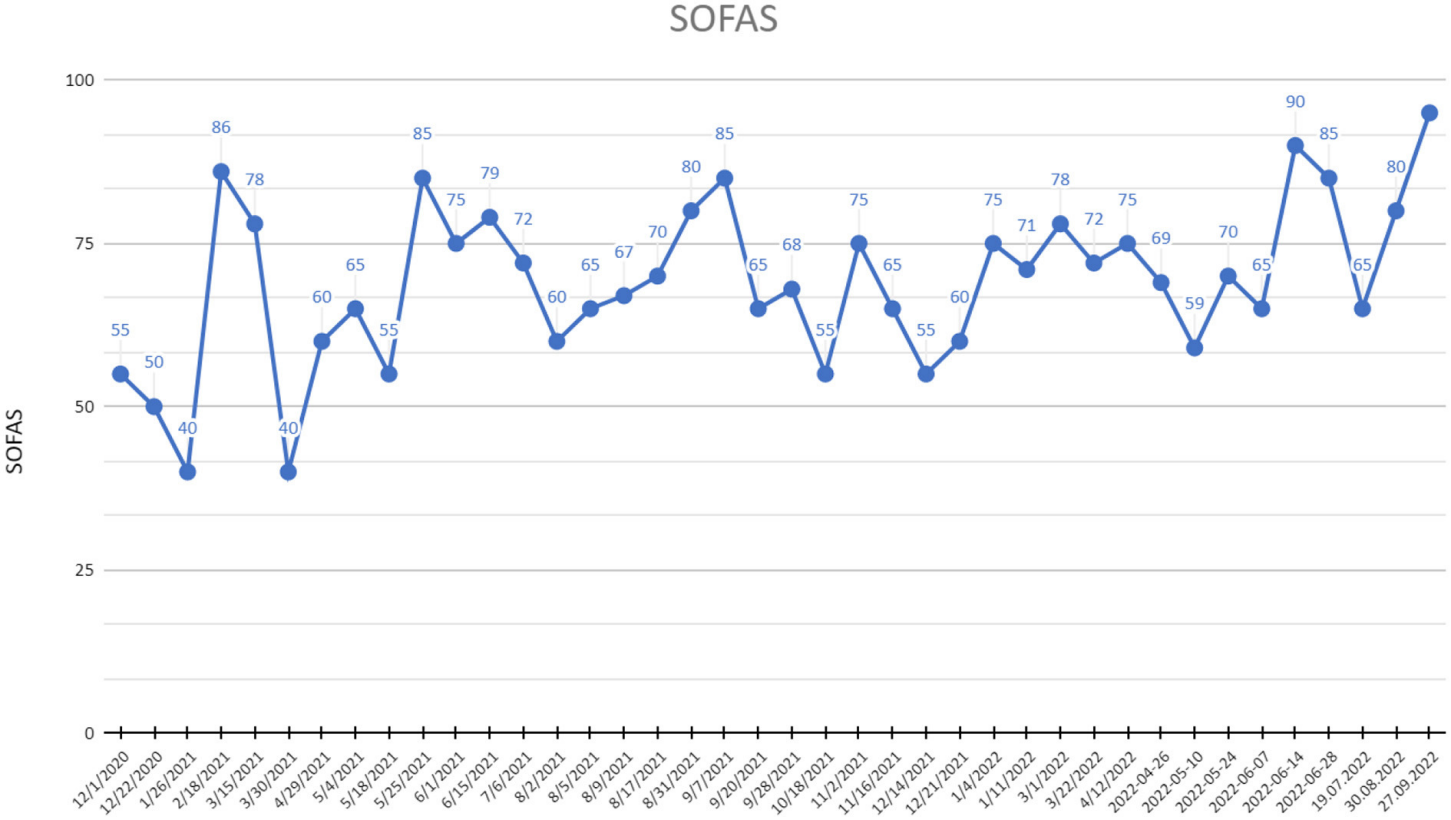
SOFAS (Social and Occupational Functioning Assessment Scale) scores at recruitment point and during 20 months of active DBS stimulation period.

Summing up, after 20 months of active DBS stimulation, the patient remained lastly in 3-month remission of MDD symptoms and a significant improvement in global functioning compared to baseline (Table 2):

— 96.55% reduction of depressive symptoms by MADRS,
— 94.4% reduction of depressive symptoms by HDRS-17,
— 87.5% reduction of anxiety symptoms (Figure 2),
— 90% increase of social and occupational functioning (Figure 3).

**Table 2:** The most important information on therapeutic intervention (MFB DBS combined with psychotherapy and pharmacotherapy) of TRD case report.

**Scales/dates**	**Baseline pre-DBSDecember 2020 **	**January 2021 2weeks of DBS **	**March 2021**	**March/April 2021—suicidal attempt **	**April 2021—1 week after discharge **	**July 2021**	**July/August 2021— blinded parameter adjustment **	**September 2021**	**October 2021**	**Early November2021 **	**Mid-November2021 **	**Late December2021**	**January 2022**	**Late April 2022 **	**Early June 2022 **	**Late June 2022 **	**Mid-July 2022**	**September 2022**
MADRS	29	39	1	39	7	8	33	3	25	3	26	11	13	28	27	1	24	0
HAMD	18	26	0		3	9	24	2	23	1	14	9	6	18	18	2	13	0
HAMA	8	11	0		2	1	7	6	11	3	9	5	6	17	13	2	8	1
SOFAS	50	40	78		60	72	60	85	55	75	65	60	71	69	65	85	65	95
BDI	22	20			12	12		16		19			18	21	26			18
SHAPS	11	11			3	12		11		5			13	14	12			6
BADS-SF	15	15			27	16		23		21			23	8	8			29
ISI	12	9			7	16		8		7			10	19	2			3
PSQI	7	5			4	9				7			8	12				10
PANAS positive	18	15			28	19				17			15	15	19			36
PANAS negative	26	21			14	17				22			26	19	20			16
Vortioxetine	10	20	20	20	20	20	20	20	20	20	10	10	10	10	20	20	20	20
Quetiapine	-	-	-	-	-	-	50	50	50	50	-	-	-	100	100	25	25	50
Bupropion	-	-	-	-	-	-	-	-	-	-	300	300	300	300	300	-	-	-
Mirtazapine	30	30	30	30	-	-	-	-	-	-	-	-	-	-	-	-	-	-
Lithium carb.	-	-	-	-	-	-	-	-	-	-	500	500	500	500	250	-	-	-
Lamotrigine	-	-	-	-	150	150	150	150	150	150	-	-	-	-	25	50	50	50
Propranolol	-	-	-	-	-	-	-	30	30	30	30	30	30	30	30	30	30	20
Escitalopram	-	-	-	-	-	-	-	-	-	-	-	-	-	-	-	-	-	-
Chlorprothixen	-	-	-	-	50 on demand	50 on demand	50 on demand	50 on demand	50 on demand	50 on demand	50 on demand	50 on demand	50 on demand	-	-	-	-	-
Promazine	-	-	-	-	-	-	-	-	-	-	-	-	-	25	25	25	25	25
DBS parameters	-	Non-specific wide stimulation of MFB area, 2.5 mA bilaterally	Disseminated field on right electrode, left side closer to MFB, 3 mA left MFB, 2.8 mA right MFB	As before	As before	As before	Electric field moved lower in the right MFB, 2.8 mA bilaterally	Amplitude reduced in right MFB to 2.5 mA due to side effects, left MFB 3 mA	Selective stimulation of MFB bilaterally left 2.4 mA, right 2.5 mA	As before	As before	As before	As before	As before	Like March 2021 but Anodic current, 3 mA left, 2.8 mA right	As before	Like March 2021, bipolar stimulation, 2.5 mA left, 2.3 mA right	As before

Scoring, medication dosage, and stimulation parameters in the course of Deep Brain Stimulation. BADS-SF, Behavioral Activation for Depression Scale-Short Form; BDI, Beck Depression Inventory; DBS, Deep Brain Stimulation; HAMA, Hamilton Anxiety Rating Scale-A; HAMD, Hamilton Depression Rating Scale 17-item version; ISI, Insomnia Severity Index; MADRS, Montgomery-Asberg Depression Rating Scale; PANAS, Positive and Negative Affect Schedule; PSQI, Pittsburgh Sleep Quality Index; SHAPS, Snaith-Hamilton Pleasure Scale; SOFAS, Social and Occupational Functioning Assessment Scale; TMS, Transcranial Magnetic Stimulation. Remission and response in MADS and HAMD in gray. Medication (mg daily).

#### 2.3.1. Psychological evaluation and psychotherapy

Shortly before the DBS implantation the patient decided to start CBT in the Psychiatry Clinic and continued until the present moment. It was considered adequate as it allowed the patient to contact the same therapist on an in-and outpatient basis. The patient is cooperative and attends weekly sessions unopposed. During acute and sudden onset of her depressive episode's interventions are supportive, focused around “getting through” the rough patch, leaving little room for long-term goals.

During those episodes the patient was largely inactive, spending time in bed and (if possible) at work with no other activity during the afternoons and weekends. She also remained extremely isolated, limiting her social contacts to forced interactions with her family and/or medical personnel, where conversations are mostly limited to yes/no answers. At the peak of those episodes the patient was anxious, restless, often reporting racing thoughts, insomnia and nightmares including realistic dreams sometimes centered around traumatic past events. On average episodes passed after 3–4 weeks, with a slow decline in anxiety and SI up to a state of slightly lowered but stable mood.

During remission or at least improvement the patient typically becomes more active and social as well as more engaged in her therapy discussing future goals, current thoughts and past events, although these changes rarely reach levels that could be considered satisfactory or expected in terms of mental health. Apart from it the patient's current psychological functioning is influenced by a high susceptibility to external stressors and very low resilience. Small external obstacles are difficult to combat for her and her self-efficacy and expected ability to cope are minimal. This is also largely influenced by her recent experiences with her illness, which she feels is entirely out of her control, leaving her with the belief of being unable to control or regulate her emotional states and decisions. However, in the course of treatment, the patient's coping abilities were observed to improve, she is to some extent able to manage her illness in a more constructive way, i.e., keep up some physical activity and manage her stress-load during low-mood episodes.

At the beginning of July 2022, the patient noted a gradual improvement in her mood, which remained stable until the end of the observation period covered in this case study (first half of October 2022). During this time the patients' symptoms were stable enough to allow her to formulate and fulfill some long-term goals, i.e., move into her own apartment and manage day -to-day tasks living alone, while working part-time as well as plan and discuss her future career options with her employer. The patient is also able to actively pursue and uphold several relationships with family and friends, including the relationship with her mother, which is now significantly improved. At the same time, for the first time since the decline of her mental wellbeing the patient is able to pursue a romantic interest and is currently involved in a relationship with a significant other.

#### 2.3.2. Additional diagnostic assessment and cognitive functioning

An evaluation of possible comorbid post-traumatic stress disorder was performed based on the ICD-10 criteria and could not be confirmed based on the current as well as previous symptoms, including onset and duration. Despite a previously acquired diagnosis of avoidant personality disorder, the current personality diagnosis (e.g., SCID-5) did not confirm this finding, nor yield a specific PD diagnosis. Immature personality traits do however emerge in the structured interview, other clinical instruments as well as observation and the patient's history. The NEO-PI-R personality inventory yielded high to very high scores in agreeableness, the anxiety and depression subscales of the neuroticism dimension as well as the subscales dutiful and deliberate of the conscientiousness dimension. Extremely low scores were found for the extraversion and openness to experience dimensions. In line with other data these results suggest extreme introversion, high negative emotionality, a tendency to deny her emotional responses coupled with low insight as well as restraint in anger responses toward others, conflict avoidance and high personal standards contributing to the observed anxiety and avoidance responses.

Neuropsychological evaluation with the use of the Cambridge Neuropsychological Test Automated Battery (CANTAB) was performed at baseline and 6 months of stimulation. The test battery included a brief cognitive safety assessment (i.e., visual memory, new learning, and reaction time), and depression-specific tasks (executive functions—spatial planning and working memory and emotion recognition). Based on the results the cognitive safety of the procedure could be confirmed by no noticeable decline in the cognitive safety assessment.

#### 2.3.3. Patient perspective

The patient filled in self-assessment questionnaires that contributed feedback including the patient's perspective. In terms of quality of life (measured by the WHOQOL-BREF questionnaire) during 20 months of DBS stimulation we have observed:

— 15.8% increase in psychological domain (from 38 to 44 points on 1–100 scale),
— 44.7% decrease in social support domain (from 56 to 31 points),
— 20.6% decrease in environmental domain (from 63 to 50 points),
— no change in physical domain (44 points).

Subjective depressive and insomnia symptoms after 20 months of active DBS compared to baseline (Table 2):

— 18.2% reduction in Beck Depression Inventory,
— 38.6% reduction in Negative Emotion and 100% increase in Positive Emotion (PANAS),
— 75% reduction of insomnia symptoms by Insomnia Severity Index (ISI; indicating no insomnia symptoms after 20 months of stimulation),
— 42.8% increase in Pittsburgh Sleep Quality Index with relatively poor sleep quality, mostly due to reported sleep fragmentation and increased sleepiness during the day, although insomnia criteria are not met,
— 53.8% decrease of anhedonia intensity in Snaith-Hamilton Pleasure Scale,
— 93.3% increase of activity in Behavioral Activation for Depression Scale-Short Form (BADS-SF),
— 100% reduction in VAS anxiety,
— 80.0% improvement in VAS mood,
— 33.3% decrease in VAS concentration,
— lack of physical discomfort (VAS).

**Figure 4:**
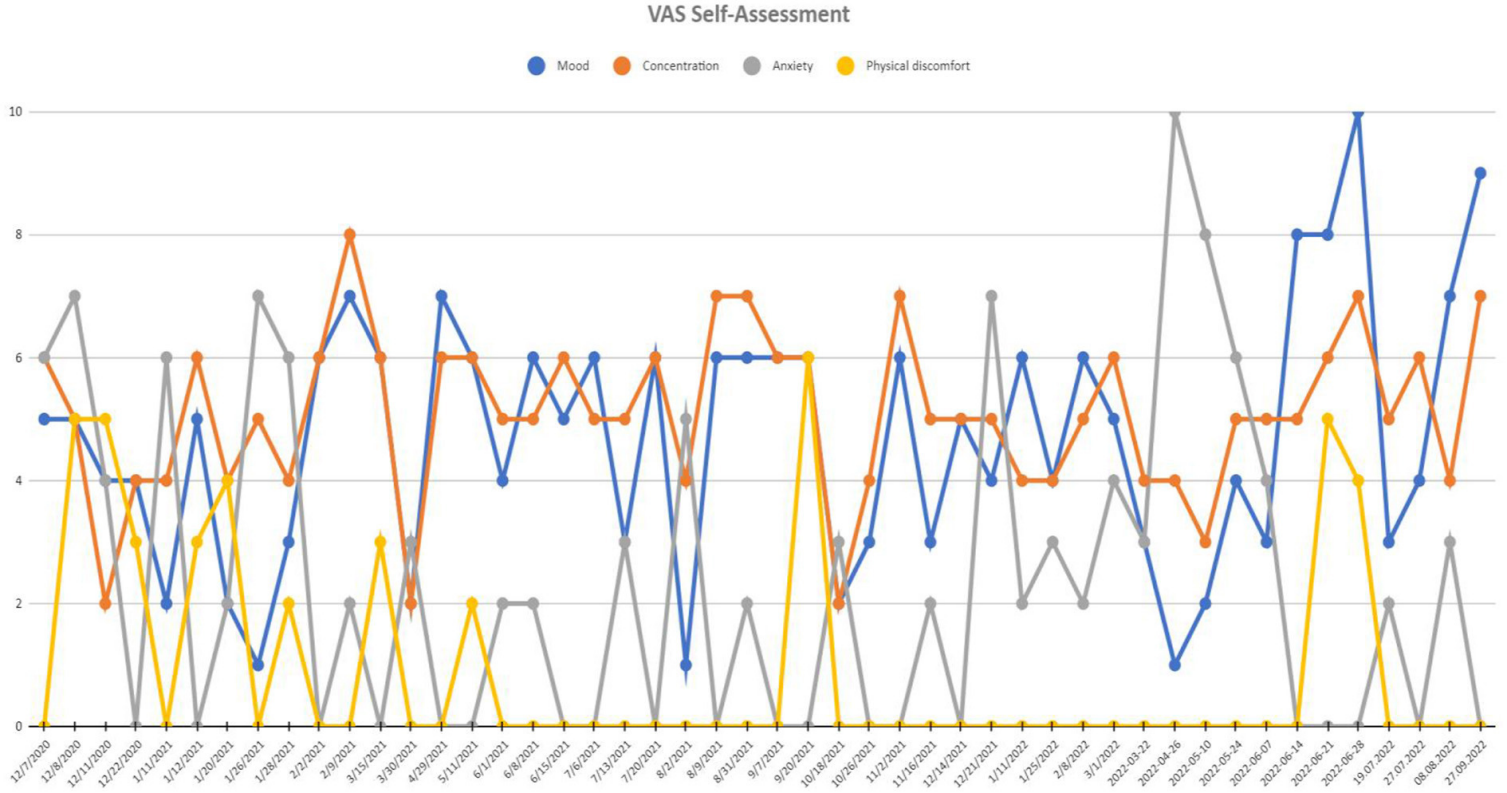
Visual analog scale (VAS) of self-assessment scores at recruitment point and during 20 months of active DBS stimulation period. Mood and concentration scales were scored positively (0-worst mood and concentration and 10-best mood and concentration), while anxiety and physical discomfort scales were scored negatively (0-least intensive anxiety and discomfort and 10-most intensive anxiety and discomfort).

**Figure 5:**
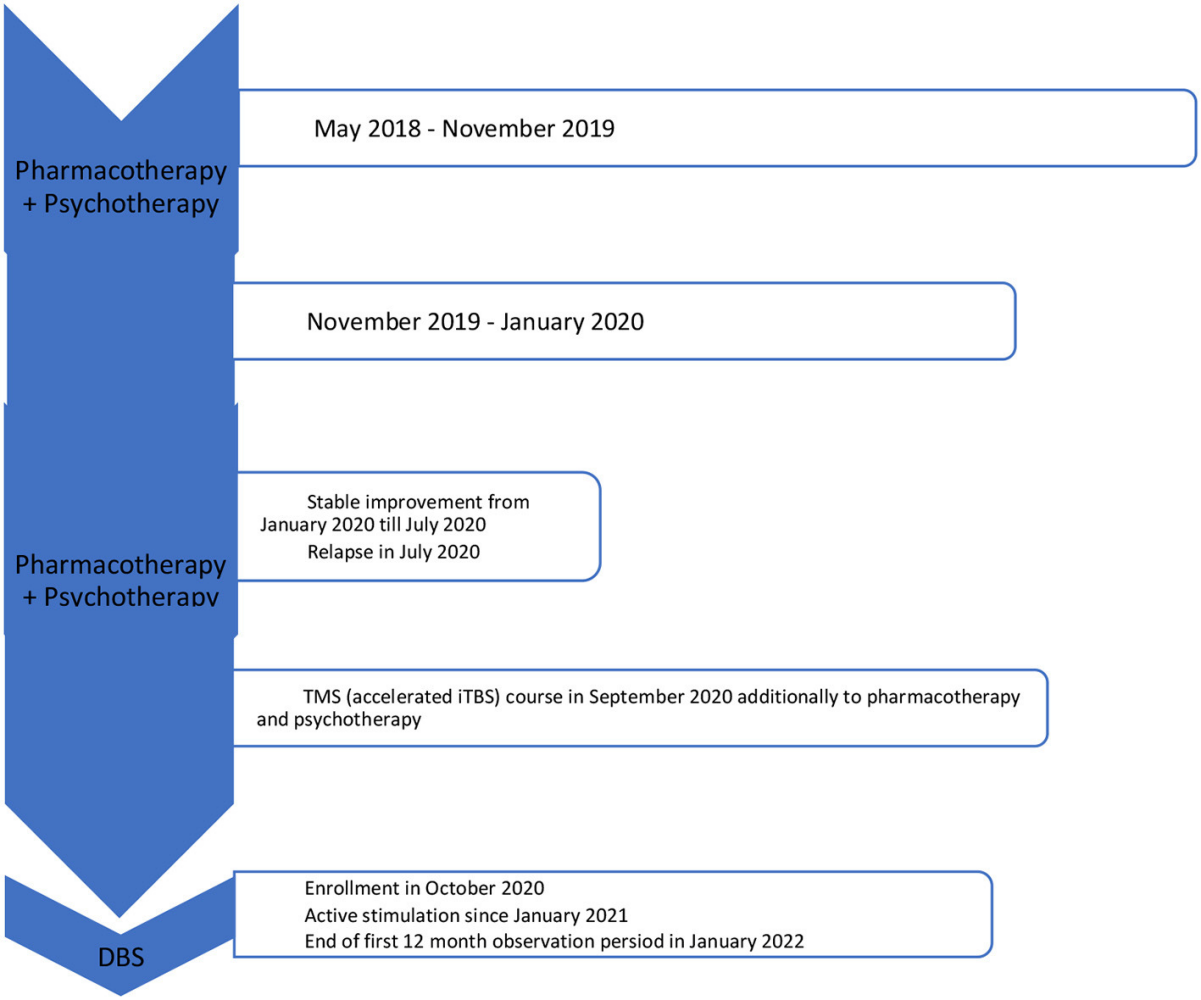
Timeline of the treatment.

See Figure 4 for more details. VAS assessment was performed during each check-up. Transient side effects of DBS were described in Section 2.3.1, all of them were eliminated by the adjustment of stimulation parameters. No side effects resulting from the location of the wires and the stimulator itself were reported. The answer to the question during the patient's last examination, “Would you have made the same decision about DBS?” was—yes, definitely yes. For detailed information on the treatment timeline, please see Figure 5.

## 3. Discussion

Within 20 months of MFB DBS subsequent remission periods have been observed with several relapses. An unambiguous interpretation of the results, especially the periods of deterioration, must take into account the psychological background as well as the external possible causes of the relapses. One rapid deterioration of the patients symptoms was observed after COVID-19 infection, which has been previously suggested by literature to exacerbate depressive symptoms (6). Another one after a scheduled change of neurostimulation and during increased impedance of C6 electrode requiring correction. Three relapses were observed without any known external causes. After 1 year of active DBS, the patient continued to report significant anhedonia (confirmed by the Snaith-Hamilton Pleasure Scale -SHAPS scores), but after the 20-month stimulation period a significant improvement was finally observed, which supports earlier reports indicating a possible positive effect on this symptom (7, 8).

Previous studies reported a rapid improvement in the 1st weeks of active MFB DBS (9, 10). After the adjustment of first stimulation parameters, also a fast remission over the course of 1 month was observed. Overall, comparing the baseline and final scores a significant response (defined as MADRS and HAMD ≥ 50%) reduction in rater-based depressive symptoms and socio-occupational functioning was found. MADRS score reduction was varying between 50 and 90%, which exceeded partially previous studies (10, 11). However, Davidson et al. reported a series of two unsuccessful MFB DBS treatments with no remission after 6 months of follow-up, defined as 50% reduction in HAMD scale (12). In presented case a 50% HAMD reduction was observed after 6 months, 66% after 12 months, and 94% after 20 months with several deterioration periods in the meantime.

According to earlier reports subcallosal cingulate DBS (SCC DBS), highest rates of clinical and functional improvements were observed after 2 years of active stimulation (13), though in case of the ventral anterior limb of internal capsule (vALIC) these parameters were comparable after 1 and 2 years of active DBS (14). In the case of MFB DBS a significant improvement in GAF scores after 1 year of stimulation was reported in a relatively large study (10).

Our case report supports MFB as an effective target of DBS, as previously demonstrated by Dandekar et al. (15). Even though some previous reports e.g., by Hitti et al. (16) using meta-regression did not reveal an optimal stimulation target to treat depression, our data suggests stimulation in the middle forebrain bundle may be an effective and well-tolerated target of surgical interventions. During the observation period the patient did not report any severe adverse effects from stimulation. The only severe incident was a suicidal attempt in March 2021, which could be considered an adverse effect of either stimulation or the MDD itself. As described by Kisley et al. suicide attempts may present as possible adverse effects but are rather connected to sham DBS than active stimulation (17).

Personality traits, low resilience and slowly improving coping mechanisms, factors not analyzed on a case-by-case basis in previous studies in the context of DBS in MDD, developed by the patient might contribute to described differences between objective and subjective assessments. From the patient's perspective, the biggest improvement was reported most often in terms of activity and anxiety reduction (considering the whole observation period).

Previous studies have not raised concerns regarding possible worsening of neurocognitive functioning, and in some studies even slight improvement was reported (18). In presented case the cognitive safety of the procedure was confirmed.

Coenen et al. reported a significant improvement in the mental health domain of quality of life after 1 year of stimulation, with no significant change in physical health domain (measured with SF-36) (10). Similarly, after 1 year and after 20 months of active DBS the patient reported an improvement in psychological domain of quality of life with no change in physical and decrease in social domain. The latter probably as a result of a worsened relationship with sister, a physician, who had been actively supporting her for the last years of treatment. Due to several hospitalizations and absences at work last year, the patient's already limited social network has noted a further decrease.

At the end of the observation period, a significant improvement of the patient's coping was reported by the psychotherapist. Meanwhile, an improvement in social and occupational functioning was observed as a consequence of the reduction of depressive and anxiety symptoms. Despite several fluctuations and periodical exacerbations of symptoms, a full remission with improvement of functioning and coping mechanisms was reached after the 20 months of active MFB DBS, especially compared to previous periods of described ECT, iTBS, and pharmacological therapies.

## 4. Conclusions

In conclusion, the presented case confirms that response and even remission levels can be reached by using MFB DBS in treatment-resistant depression. Nevertheless, personalization of every combined therapy with DBS is necessary. Thanks to close cooperation with the psychologist, a broad assessment of dynamics of this improvement was possible. DBS of treatment-resistant patients requires constant objective and subjective evaluation and adjustment, which has finally led to the remission of clinical symptoms and high functioning level of reported TRD patient. The presented case report further underlines the need for studies exploring individual factors (including past traumas), psychological traits (personality traits, coping strategies, and level of resilience) as well as comorbid diagnoses as factors influencing the response to DBS in patients with treatment resistant depression. More reports on long-term observations in DBS treatment in TRD trials (especially focused on MFB target) are needed.

## Data availability statement

The raw data supporting the conclusions of this article will be made available by the authors, without undue reservation.

## Ethics statement

The studies involving human participants were reviewed and approved by Wroclaw Medical University Ethics Committee. The patients/participants provided their written informed consent to participate in this study. The patient signed a permission for the publication of this Case Report.

## Author contributions

JR was responsible for psychiatric conceptual work, supervision, review over the psychiatric part of the manuscript text, ethical approval, and funding. TW was responsible for drafting the first version of the manuscript, data extraction and analysis, and database management. KF-W was responsible for the psychological evaluation and psychotherapy section of the manuscript. PP was responsible for the pharmacotherapy section of the manuscript. KS and AW were responsible for the neurosurgical section of the manuscript. PT was responsible for the neurosurgical conceptual work, data collection, supervision of the neurosurgical part of the manuscript, ethical approval, and funding. All authors contributed to manuscript revision, read, and approved the submitted version.
